# Exploring the marketing environment for maize seed in Kenya: how competition and consumer preferences shape seed sector development

**DOI:** 10.1080/15427528.2020.1737296

**Published:** 2020-03-09

**Authors:** Pieter Rutsaert, Jason Donovan

**Affiliations:** a Socioeconomics Program, International Maize and Wheat Improvement Centre (CIMMYT), Nairobi, Kenya; b Socioeconomics Program, International Maize and Wheat Improvement Centre (CIMMYT), Texcoco, Mexico

**Keywords:** Africa, agro-dealers, maize, new product development, seed preferences, varietal turnover

## Abstract

During the past decade, sizable investments have been made to strengthen maize (*Zea mays L*.) seed production in Eastern and Southern Africa by private seed companies. However, efforts have generally overlooked downstream issues, such as how seed companies market their products and position their business in a competitive market. This paper assesses competition and customer preferences in Kenya at the retail level for varieties from the parastatal, private domestic and international companies. Data were collected from agro-dealer surveys (n = 80) and farmer intercept interviews (n = 377). Compared to the market leader, the parastatal Kenya Seed Company, private domestic and international companies provide greater value to farmers by selling varieties that are, on average, 10 years younger. However, these companies offer few late-maturing varieties, thus giving the parastatal a near monopoly in that market segment. Kenya Seed Company also excels at being present at all sales locations with their varieties. If private domestic companies focus more on smallholders with lower budgets (including travel budget), they should consider the most cost-effective ways to extend their networks to remote areas as well as compete with the lower prices of Kenya Seed Company. Modern breeding programs should explicitly consider these marketing challenges when designing strategies for seed production and engagement with seed companies.

## 1. Introduction

Strengthening in-country seed production forms a key element of strategies for advancing food security and poverty reduction goals (Lipton and Longhurst 1989; Byerlee and Eicher 1997; Louwaars and de Boef 2012), as well as for mitigating the effects of climate variability and extremes (Access to Seeds Foundation 2019; Atlin, Cairns, and Das 2017; Fisher et al. 2015). While different philosophies and pathways exist on what goals and means should be employed to advance seed sector development (McGuire and Sperling 2016), for the maize seed sector in East and Southern Africa, the dominant approach for maize emphasizes seed production by privately owned, often small and medium-scale, businesses and agro-input companies (AGRA 2017; McGuire and Sperling 2016; Toenniessen, Adesina, and DeVries 2008; Erenstein and Kassie 2018).

Kenya provided an early success story of public-sector maize-breeding programs (Gerhart 1975; De Groote et al. 2002). Since the liberalization of the Kenyan seed market in the mid-1990s, the country has experienced an expanding offer of hybrid maize seed. Before the liberalization, seed was provided through the parastatal Kenya Seed Company (KSC). In the years following liberalization, they continued to dominate the seed market. At the start of the twenty-first century, the first international companies (IC), including Monsanto and Pioneer as well as Pannar, a South African seed company, began selling maize seed in Kenya (Swanckaert 2012). Around the same time, private domestic companies (PDC) started selling seed in Kenya. In 2015, 19 companies were producing and selling maize seed in Kenya, including 8 IC, 10 PDC, and the parastatal KSC (The African Seed Access Index 2016). However, the liberalization of the seed market has not been without challenges. KSC continues to dominate the maize seed market (Nagarajan, Naseem, and Pray 2019; Tripp and Rohrbach 2001; Smale and Olwande 2014) and sells seed at fixed low price regardless of package, type (hybrid or open-pollinated), or place (center of the city or rural market) (Nambiro, de Groote, and Osura 2002). Expensive and long varietal registration and release processes still hamper competition by restricting the launch of new seed products in the Kenyan market (Spielman and Smale 2017).

A key expectation of PDC, as compared with their multi-national counterparts, is that they are more willing to seek out traditionally underserved segments of the seed market, including poor farming households located in less-favored production zones. For this reason, these businesses have been the preferred partner of governments, international research and donor organizations for production and diffusion of improved varieties emerging from public-sector maize-breeding programs. The expansion of the maize seed supply by locally owned businesses in the Kenya market, as well as other markets in the region, has been supported by long-term donor engagement aimed at strengthening the breeding work by public-sector agencies (e.g., providing access to new germplasm, building capacity of breeders, implementing on-farm trials). At the same time, investments have also aimed to strengthen the capacity of domestic seed businesses to produce and market quality seed and renew their seed portfolio. Through breeding projects by CIMMYT and partners, including Water Efficient Maize for Africa (WEMA), Drought Tolerant Maize for Africa (DTMA), Improved Maize for African Soils (IMAS), Stress Tolerant Maize for Africa (STMA), >80 stress-tolerant varieties were released in Kenya in the last 15 years. Through the program for Africa’s Seed Systems (PASS), AGRA invested significantly in breeding, training, and production programs of seed companies in Kenya (AGRA 2017).

However, the focus of development interventions in formal maize seed systems has concentrated mainly on seed production and less on understanding how PDC, some of which are likely to be flush with new seed products, can market their new seed products and position their business vis-à-vis the market leader – KSC, and growing competition from IC. Farmers are now presented with different choices for seed purchase, but likely have limited information at hand on the attributes of these products (Waldman et al. 2017). But how successful are companies in understanding their target audience and what that audience needs with respect to maize seed? By taking a snapshot of seed sales in Kenya at the retail level, i.e., by looking at both seed availability and farmer preferences, the paper aims to understand the positioning of PDC in a competitive market and provide recommendations for further strengthening of a dynamic and strong local seed sector.

The remainder of this paper is organized as follows: In Section 2, current frameworks in seed systems are discussed and complemented with concepts from new product development. In Section 3, the two data collection methods, i.e., the agro-dealer survey and farmer intercept interviews, are discussed and Section 4 reports the results. In the final section, we discuss the implications of the competitor and customer analysis for the local seed sector as well as for donors and implementing partners supporting this sector.

## 2. Seed systems framework

Frameworks for assessing formal maize seed systems have centered largely on seed production and distribution – with the major issue being getting enough quality seed to farmers. In his conceptualization of the maize seed industry development, Morris (1998) discussed a pathway of four stages through which a seed industry evolves, going from “preindustrial stage” to “maturity stage.” Indicators to evaluate the industry stage are mainly based on production criteria, including the seed production and distribution capacity (e.g., capacity to produce hybrids). A similar analysis of the growth stages of the seed industry in sub-Saharan Africa was recently carried out by Ariga et al. (2019). In their synthesis of seed systems for vegetatively propagated crops, Bentley et al. (2018) highlighted three key dimensions: availability, access, and quality. Langyintuo et al. (2010) assessed the bottlenecks for maize seed business growth and expansion in Eastern and Southern Africa. Besides several supply-side-related challenges, such as company establishment and seed production, they also considered demand-side problems in seed systems, including low adoption rates, lack of demand estimates and limited reach of extension. However, a common assumption in these frameworks and assessments is that seed companies operate in a quasi-monopolistic system, where increased supply translates into increased sales and market competition can be discarded.

Recent trends in the Kenyan maize seed sector beg the question: what if farmers that are looking for improved maize varieties all have access to an existing seed portfolio? This would imply that new seed products would need to capture market share from existing players, a challenge that has received a substantial amount of attention in the field of new product development (Gray et al. 2004; Cooper 1986, 2011; Krishnan and Ulrich 2001; Boehlje, Roucan-Kane, and Broring 2011) but not in seed systems. In general, launching a new seed product poses considerable risk and expense for seed businesses. Farmers have limited information on seed attributes (Simtowe et al. 2019) and tend to be adverse to experimentation in seed utilization (Fisher and Kandiwa 2014; Das et al. 2019). These challenges are compounded by weak agricultural extension programs and limited engagement by agro-dealers with their farmer clients at the point of sale (Rutsaert and Donovan 2020). Moreover, we know little about the reach or effectiveness of seed demonstration plots, a key tool employed by maize seed companies to build demand for new products.

Incorporating concepts and frameworks of new product development and innovation literature can support the local seed sector in their business decisions. When a seed company launches a new product or targets a new area, it can be seen as an innovation in the local offer for farmers. Christensen and Raynor (2003) make the distinction between three types of innovation: (i) sustaining innovations focused on new attributes added to existing products (for example, releasing a new hybrid that has a higher yield or stress resilience than existing hybrids in the market), (ii) new market innovations that offer completely new products (releasing hybrids in an area where only saved seed is being used) and (iii) low-end innovations focusing on less demanding customers and delivering a product at lower cost (selling a cheaper open-pollinated variety (OPV) in a hybrid-dominated market). These different innovations require very different approaches in targeting customers, creating value, dealing with competition and getting profit (Gray et al. 2004). Understanding the type of innovation, a seed company wants to introduce, can guide decision-making on the criteria mentioned above. In the case of Kenya, where most farmers buy hybrid seeds (Smale and Olwande 2014) and OPVs have been phased out in the main maize-producing zones (Rutsaert and Donovan 2020), sustaining innovations in the formal maize seed sector is most prevalent.

Incorporating a stronger demand-side perspective into formal maize seed systems requires new thinking about the role of market structure and competition in shaping the opportunities and constraints for seed businesses. Two key aspects of new product development, according to Gray et al. (2004), are customer analysis and competitor analysis. An important element of customer analysis is understanding the value a new product gives to the customer, also referred to as consumer surplus. For sustaining innovations, this can be done by improved performance on highly valued attributes. Knowing which customers are interested is also critical for the success of sustaining innovations. It is often the more demanding and knowledgeable customers, who will be the first adopters of these new, improved products (Simtowe et al. 2019). Besides knowing who their customers are, seed companies should also be aware of their competitors. Competitor analysis and the concept of competitive advantage are fundamental concepts in the corporate world and business-management literature (Peteraf 1993; Barney 1991; Porter 2008, 1980). In his Five Forces Model, Porter (1980) provides a framework for competitor analysis, focusing on (i) new entrants, (ii) substitute products, (iii) bargaining power of suppliers, (iv) bargaining power of buyers and (v) rivalry among existing firms, i.e., the direct competition in a sector. Access and understanding of market data are required to have a better understanding of competition and customers and this is something that is lacking in maize seed markets. In his paper on the upcoming reforms of the CGIAR, Haddad (2020) underscored the importance of research on consumer insights as one of the key gaps in public research. While he was referring to food systems in the paper, we argue the same argument holds true for seed systems. This paper aims to fill this gap by focusing on seed retail to assess the current competition domestic seed companies are facing and by getting a better understanding of the customer (farmer) buying maize seed, either from Kenya-based companies, international competitors, or KSC.

## 3. Material and methods

### 3.1. Data collection

Data were collected in Kenya at the start of the 2019 main maize-growing season that began in March and ended in June. Structured surveys were carried out with agro-dealer attendants, to include store owners, managers or full-time employees, and intercept interviews were implemented with farmers who visited agro-dealerships to purchase maize seed. Important to note is that the majority of maize seed in Kenya is purchased within a short period, roughly 2–3 weeks, following the first rains, which mark the start of the planting season. Because the tropical cyclone “Idai” redirected rains away from East Africa (FAO 2019), the traditional starting period of the maize-growing season was disturbed by scattered and absent rainfall. We adjusted our data collection to weather patterns and carried out intercept interviews in locations where rain had started to fall, as farmers were only buying during those days.

### 3.2. Agro-dealer survey

**Figure 1.**
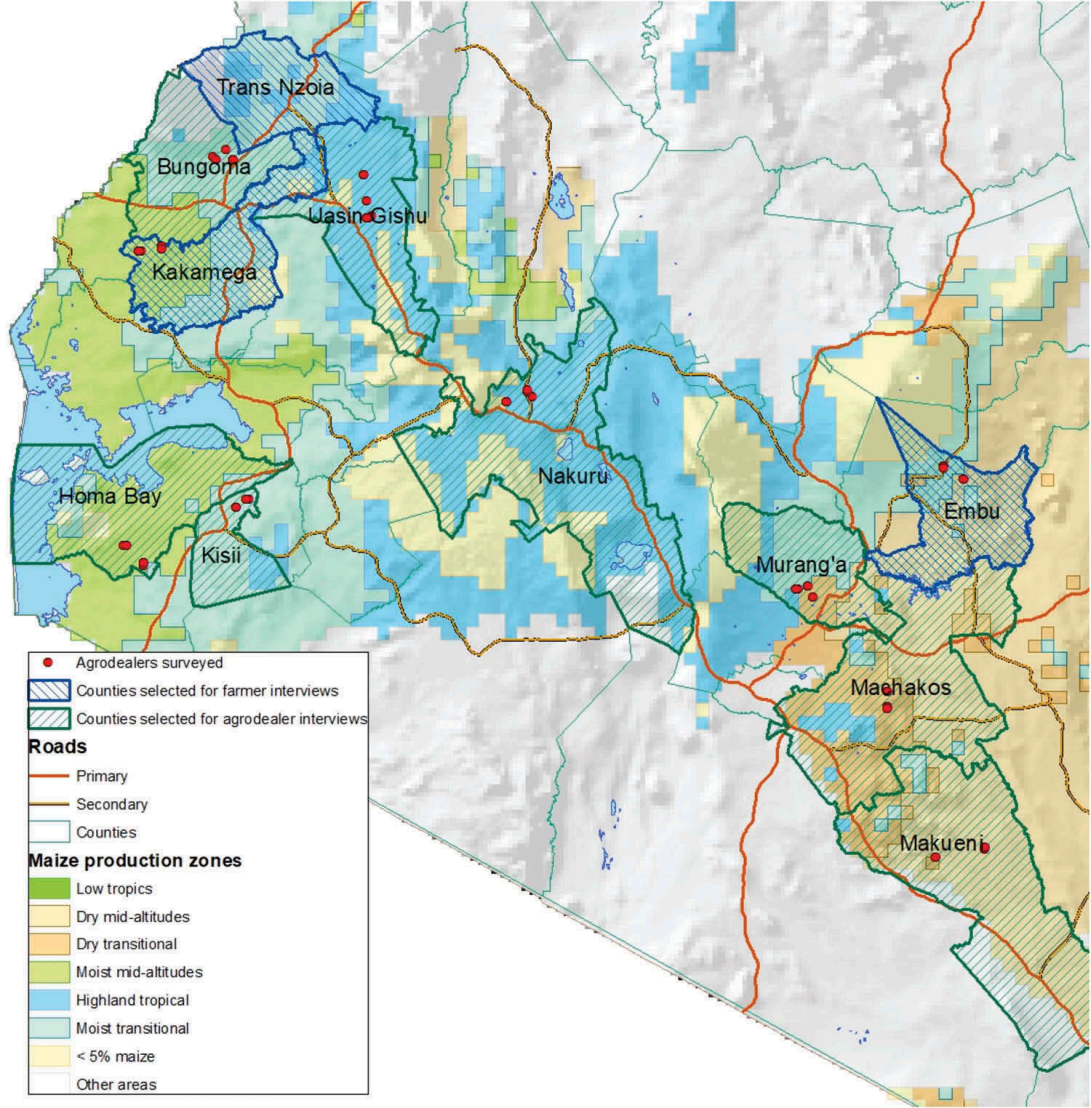
Locations of agro-dealer surveys and farmer intercept interviews.

A structured survey was carried out with 80 agro-dealers, where sample design considered potential differences among agro-dealers with respect to the agro-ecological zone in which they were located and their proximity to urban centers. Two counties were randomly selected in each of the following agro-ecological zones: tropical highlands, moist transitional, moist mid-altitude, dry transitional and dry mid-altitude. In these 10 counties, one sub-county was randomly selected and in that sub-county, four agro-dealers in the sub-county main center (referred to as urban center) and four agro-dealers in rural centers were randomly selected from a centralized agro-dealer database of Kenya. This database was verified and adjusted where necessary by local government officials at the sub-county level. Figure 1 represents the selected counties and locations of the agro-dealers. Interviews lasted roughly 60 minutes and information was recorded with electronic tablets by experienced enumerators. The survey was extensively pretested before the seed-selling season and revised across several iterations. Interviews were carried out with the individual, who was most knowledgeable about maize seed sales and day-to-day transactions. Most often, the respondent was the store owner but, in some cases, it was a store manager or experienced employee.

### 3.3. Farmer intercept interviews

Intercept interviews were carried out with farmers exiting the selected agro-dealers. Selected farmers had purchased maize seed for themselves or someone in their family and reported to be involved in maize farming. Examples of those excluded from intercept interviews included drivers who picked up seed for their employers, as well as family members who purchased seed, but were not involved in maize farming. Respondents were invited to participate when coming out of the agro-dealer in a 10- to 15-min survey. Tents were placed in the vicinity of the agro-dealer to provide shade during interviews, as well as to reduce potential unease (and bias) among respondents answering sensitive questions about seed selection inside the agro-dealership. Two teams of three enumerators carried out the survey. A team leader recruited farmers coming out of the store, who made a purchase and two enumerators carried out the surveys. Respondents received a small incentive for their participation. Each farmer coming out of the agro-dealer was invited to participate in the survey.

Three counties were selected on the basis of their agro-ecological zone and the high prevalence of maize farming: Embu (dry mid-altitude), Kakamega (moist mid-altitude), and Trans-Nzoia (highlands) (see Figure 1). However, the results of the intercept interviews in Trans-Nzoia were not included in the analysis for two reasons: (i) sample size was small because the absence of rain and (ii) KSC dominated the retail landscape, with little competition from other seed companies. In each county, the main county center and a rural location were selected and depending on store traffic per agro-dealer, one to three agro-dealers were chosen in each location to recruit farmers. Pretesting was done in the first week of the maize seed-purchasing season in Kiambu county, where early rains had resulted in an early wave of maize seed purchases.

### 3.4. Data analysis

The focus of the analysis, at agro-dealer and farmer levels, was on the varieties offered by agro-dealers and purchased by local farmers, respectively. To evaluate the performance of the parastatal KSC, private domestic companies (PDC) and international companies (IC), all varieties captured in our sample were classified in one of the three categories. Data were further analyzed on a category level; examination of distinct varieties or company performance was not the focus of this study. Statistical analyses were performed with SPSS 22.0 (SPSS Inc., Chicago, IL, USA) and STATA 15.0. Statistical significance was evaluated at α = 0.05. We carried out descriptive statistics, such as Chi-square association tests, independent sample t-tests, and one-way analysis of variance (ANOVA) to compare variety attributes and consumer profiles between parastatal, national, and domestic companies as well as characteristics of farmer samples in Embu and Kakamega.

### 3.5. Sample description

Table 1 contains basic information on the selected agro-dealerships (n = 80). The selected agro-dealerships had, on average, 8 years in business at the time of data collection and most stores were owned by a sole proprietor. A few dealerships were direct agents of seed companies, KSC in most cases. Approximately half of the stores reported the sale of maize seed as one of the top three annual revenue generators and it was the most important annual revenue generator for roughly 10%.

**Table 1.** Characteristics of the agro-dealers participating in the survey.

	Total
N	80
Years in business	8.3 (6.7)
Type of ownership (%)	
Sole proprietor	92.5
Partnership	5.0
Cooperative	2.5
Agent of a seed company (%)	15.0
Importance of maize in revenue (%)	
Most important	10.0
In top 3	51.3
Not in top 3	48.7

Standard deviation in parentheses

The characteristics of the farmer sample participating in the intercept interviews in Kakamega (n = 205) and Embu (n = 172) are described in Table 2. Roughly one-third of our sample consisted of female farmers in both locations with an average age was close to 50. Education levels were lower in Kakamega, with almost 70% not having finished secondary education, compared to approximately 50% in Embu. Maize fields were small in both locations, with an average of 0.61 ha; the share of maize sold was higher in Embu. Public transport was the most used transport to purchase maize in both locations.

**Table 2.** Characteristics of the farmers participating in the intercept interviews.

	Kakamega	Embu	*t*-Value/Chi-square	*p*-Value
N	205	172		
Gender (%)			0.874	0.350
Male	66.8	62.2		
Female	33.2	37.8		
Age in years	46.9 (15.4)	49.9 (14.5)	−1.957	0.051
Education level (%)			18.534	<0.001
Higher than secondary	10.7	25.0		
Secondary	20.5	26.2		
Lower than secondary	68.8	48.8		
Farming experience in years	17.8 (14.6)	18.2 (14.1)	−0.260	0.795
Size of maize field in acres	1.4 (1.4)	1.5 (2.2)	−0.140	0.888
Percentage of maize harvest sold	26.9 (29.3)	43.3 (37.5)	−4.659	<0.001
Means of transport (%)			12.779	0.005
Own transport (car, motorcycle)	17.1	25.6		
Public transport	70.7	57.6		
By foot or bicycle	12.2	16.9		
Cost of traveling in USD	0.67 (0.74)	0.50 (0.69)	2.322	0.021
Visited urban agro-dealer (%)	54.1	57.6		

Standard deviation in parentheses.

1 USD = 99.9 KES at the start of the data collection, 18 March 2019.

## 4. Results

### 4.1. Competitor analysis

In total, 59 different varieties were available in the agro-dealerships (Table 3) for the 2018 maize seed-purchasing season, of which 15 were produced by KSC, 20 were produced by PDC and 24 by IC (including Monsanto Seed Co, Pioneer, Syngenta, and Pannar). The average varietal age (based on release date^1^) produced by PDC and IC was around 11 years; varieties coming from KSC were significantly older, with an average varietal age of 21 years. Whereas a large part of the offer by KSC was focused on late-maturity varieties, this was not the case for PDC and IC. The focus of PDC was largely on intermediate-maturity varieties, while IC had a good share of early-maturity varieties.

**Table 3.** Variety offer in Kenya at the agro-dealer coming from Kenya Seed Company, private domestic companies, and international companies (n = 80).

	Total	KSC	PDC	IC
Different varieties	59	15	20	24
		25.4%	33.9%	40.7%
Average variety release age	14.0 (7.1)	21.2 (7.8)	11.6 (4.2)	11.6 (5.4)
Maturity levels (%)				
Early maturity variety	22.6	13.3	18.2	32.0
Intermediate maturity variety	51.6	33.3	68.2	48.0
Late maturity variety	25.8	53.3	13.6	20.0

Standard deviation in parentheses.

Table 4 contains an overview of varieties from the three company categories available at agro-dealers across the maize-growing area. Noteworthy is the difference in the physical accessibility of varieties. Although PDC produced a significant share of the varieties available in Kenya, access to these varieties seemed to be problematic. They accounted for only 12% of stock-keeping units (SKUs) or distinct items for sale available at our sampled agro-dealers and there were no varieties of PDC available in 55% of the agro-dealers. The spread across different agro-ecological zones is most equally divided for the IC, except for a lower presence in the highlands. Varieties from KSC were mostly available in the highlands and wet upper mid-altitude, which reflects its focus on late-maturity varieties. Varieties from PDC were mainly available in the wet-lower and upper mid-altitude zones; availability in the dry zones was low.^2^

**Table 4.** Availability of varieties of Kenya Seed Company, private domestic companies, and international companies at the agro-dealer (n = 80).

	Total (n = 640)	KSC (n = 309)	PDC (n = 78)	IC (n = 253)	*F* value/Chi-square	*p*-Value
Share of stock keeping units (SKUs) (%)		48.3	12.2	39.5		
No presence in store (%)		2.5	55.0	7.5		
Agro-ecological zone (%)					135.734	<0.001
Dry mid-altitude	16.3	10.4	7.7	26.1		
Dry transitional	13.8	9.7	9.0	20.2		
Wet Lower Mid-altitude	23.9	13.6	46.2	29.6		
Wet Upper Mid-altitude	26.4	34.3	29.5	15.8		
Highland	19.7	32.0	7.7	8.3		
Supplier (%)					20.003	<0.001
Seed company	27.0	34.6	26.9	17.8		
Local distributor	73.0	65.4	73.1	82.2		
Reliability of supply (%)					17.467	0.002
Very reliable	70.5	75.1	65.4	66.4		
Averagely reliable	23.9	21.7	20.5	27.7		
Not reliable	5.6	3.2	14.1	5.9		
Pack size					35.559	<0.001
1 or 2 kg	93.0	86.7	98.7	98.8		
10 or more kg	6.9	13.3	1.3	1.2		
Average retail price in USD	2.20 (0.34)	1.95^a^ (0.28)	2.33^b^ (0.23)	2.47^c^ (0.18)	348.3	<0.001
Average retail margin in USD	0.17 (0.11)	0.16^a^ (0.10)	0.18^a,b^ (0.10)	0.18^b^ (0.12)	3.626	0.027

Standard deviation in parentheses.

The a-c indicates significantly different average scores using ANOVA and Scheffe post-hoc tests (when equal variances not assumed: Dunnett’s C post-hoc test).

1 USD = 99.9 KES at the start of the data collection, 18 March 2019.

**Figure 2.**
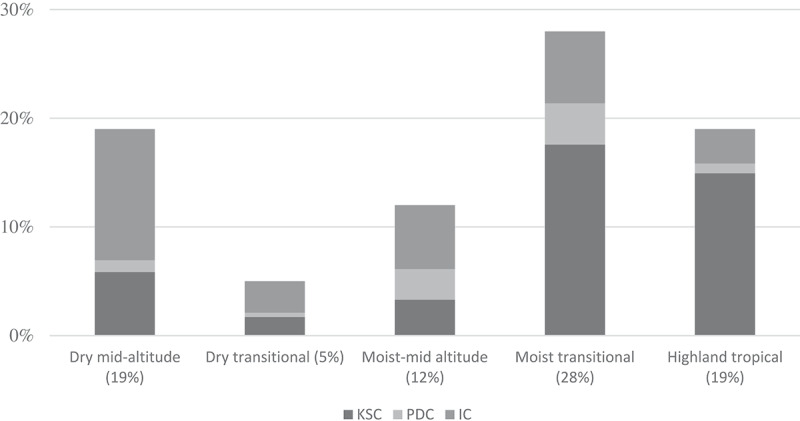
Presence of maize seed products at agro-dealers from Kenya Seed Company, private domestic companies, and international companies per agro-ecological zone, weighted by the relative size of maize production area.

Figure 2 is meant to provide a deeper look into retail presence per agro-ecological zone, with each zone weighted by the share of maize production. KSC dominated the offer of maize varieties in the maize belt, i.e., the highlands and moist transitional zone, where late-maturing varieties were most popular. In both dry zones, IC had a strong presence. Varieties of PDC were mostly present in the moist mid-altitude and moist transitional zones.

**Figure 3.**
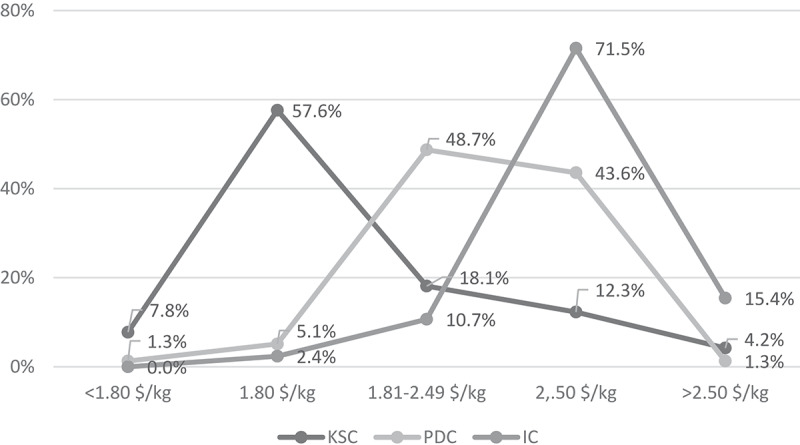
Price points at the agro-dealer of seed from Kenya Seed Company, private domestic companies, and international companies (n = 640).

Local distributors (large agro-dealers or wholesalers) provided three out of four SKUs to the sampled agro-dealers and this percentage was highest for IC, followed by PDC. The reliability of supply was different between the different company categories, with the parastatal outperforming all others. Unreliable supply was highest among the PDC. Most seed in the Kenyan market was sold in two-kg packs; only KSC sold it in larger packs. Price points of varieties also differed significantly among the three. Figure 3 contains more detail about the price setting of the different producing groups. Varieties sold by KSC were priced at 1.80 $/kg^3^ (= 180 KES/kg) or sometimes lower when sold in bags larger than 2 kg. IC favored the 2.50 $/kg (= 250 KES/kg) as price point, almost 40% higher than the parastatal price, with >70% of varieties sold at that price. The pricing strategy of PDC was less discernible. Some opted to sell their seed at a similar price as their international competitors, others maintained the price below the 2.50 $/kg threshold. The difference in sales price did not result in large margin differences for the agro-dealers. The average margin per kg was 0.17 USD (or 7.5% of sales price), being slightly lower for the parastatal varieties because of lower unit margin per kg for high-volume packages of 10 kg and 25 kg.

### 4.2. Customer analysis

Seed purchases by farmers, collected through intercept interviews at the agro-dealers in Kakamega and Embu are shown in Tables 5 and 6, respectively. In Kakamega, KSC varieties were most popular, accounting for 65% of maize seed sales during the monitoring period. Sales from PDC accounted for almost 20% and varieties from IC 15%. A different story emerged in Embu, where >55% of the purchases represented varieties from IC. Only three farmers bought a variety from a PDC; therefore, we left that category out of the data analysis from Embu. The price levels were in line with results reported on access in Table 4, with clear differences in price categories in Kakamega. In Embu, however, prices of varieties from KSC were higher than those in Kakamega, exceeding the 1.80 $/kg benchmark. Farmers in Kakamega mostly purchased late-maturity varieties (from parastatal) and intermediate-maturity varieties (from PDC), while they preferred early-maturity varieties (from IC) and intermediate-maturity varieties (from KSC) in Embu. Seed of IC was mainly purchased at urban agro-dealerships in both locations; the parastatal varieties were preferred in rural areas. Travel cost of farmers preferring varieties of IC was in both places significantly higher than that of farmers purchasing varieties from KSC.

**Table 5.** Farmer seed purchases of Kenya Seed Company, private domestic companies, and international companies at agro-dealers in Kakamega (n = 205).

	Total (n = 217*)	KSC (n = 142)	PDC (n = 41)	IC (n = 34)	*F* value/Chi-square	*p*-Value
Market share (%)		65.4	18.9	15.7		
Different varieties		8	9	9		
Average price in USD (SD)	2.00 (0.30)	1.80^a^ (0.08)	2.29^b^ (0.21)	2.48^c^ (0.10)	602.947	<0.001
Maturity level (%)					198.734	<0.001
Early	6.9	0.0	2.4	41.2		
Intermediate	31.3	9.9	95.1	44.1		
Late	61.8	90.1	2.4	14.7		
Agro-dealer location					38.858	<0.001
Urban	55.8	41.5	70.7	97.1		
Rural	44.2	58.5	29.3	2.9		
Cost of traveling in USD (SD)	0.70 (0.75)	0.55^a^ (0.57)	0.83^a,b^ (0.99)	1.15^b^ (0.86)	10.538	<0.001
Socio-demographics of buyers						
Gender (%)					3.836	0.147
Male	68.2	66.2	63.4	82.4		
Female	31.8	33.8	36.6	17.6		
Age (SD)	47.1 (15.2)	47.9 (16.1)	45.9 (13.5)	44.9 (13.2)	0.685	0.505
Size of maize field in acres (SD)	1.5 (1.5)	1.4 (1.5)	1.6 (1.4)	2.0 (1.6)	2.396	0.094
Seed volume purchased in kg (SD)	10.7 (9.2)	10.5 (8.8)	10.7 (11.5)	11.2 (8.1)	0.085	0.919
Part of maize harvest sold in percentage (SD)	28.4 (30.0)	29.4^a,b^ (29.6)	18.4^a^ (26.5)	36.1^b^ (33.1)	3.540	0.031
Years growing same variety (SD)	4.6 (5.8)	3.6 (6.3)	2.3 (2.5)	1.7 (2.0)	2.163	0.119
Key attribute of the variety					37.358	0.011
High yield	61.5	66.7	46.3	58.8		
Early maturity	14.6	6.5	36.6	20.6		
Good stalk quality	8.0	8.7	7.3	5.9		

Standard deviation in parentheses.

The a-c indicates significantly different average scores using ANOVA and Scheffe post-hoc tests (when equal variances not assumed: Dunnett’s C post-hoc test).

*12 farmers bought two varieties of different categories and are therefore included in both categories for the analysis of socio-demographics.

1 USD = 99.9 KES at the start of the data collection, 18 March 2019.

**Table 6.** Farmer seed purchases of Kenya Seed Company and international companies at agro-dealers in Embu (n = 172).

	Total (n = 181*)	KSC (n = 77)	IC (n = 104)	*t*-Value/Chi-square	*p*-Value
Market share (%)		41.8	56.5		
Different varieties		11	10		
Average price in USD (SD)	2.31 (0.29)	2.01 (0.16)	2.52 (0.11)	−25.277	<0.001
Maturity level (%)				81.175	<0.001
Early	44.2	9.1	70.2		
Intermediate	40.9	55.8	29.8		
Late	14.9	35.1	0.0		
Agro-dealer location (%)				19.966	<0.001
Urban	58.0	39.0	72.1		
Rural	42.0	61.0	27.9		
Cost of traveling in USD (SD)	0.49 (0.69)	0.36 (0.43)	0.59 (0.82)	−2.394	0.018
Socio-demographics of buyers					
Gender (%)				0.461	0.497
Male	60.8	63.6	58.7		
Female	39.2	36.4	41.3		
Age (SD)	50.2 (14.6)	50.9 (16.1)	49.7 (13.4)	0.524	0.601
Size of maize field in acres (SD)	1.5 (2.2)	0.94 (1.2)	2.0 (2.6)	−3.581	<0.001
Seed volume purchased in kg (SD)	9.7 (13.9)	6.2 (9.9)	12.2 (15.7)	−3.145	0.002
Part of maize harvest sold in percentage (SD)	44.9 (37.2)	31.4 (36.6)	54.9 (34.6)	−4.375	<0.001
Years growing same variety (SD)	5.1 (5.1)	5.5 (6.1)	4.77 (4.3)	1.641	0.104
Key attribute of the variety				15.486	0.078
High yield	61.2%	64.9%	57.3%		
Early maturity	14.8%	15.6%	14.6%		
Drought resistance	10.4%	2.6%	16.5%		

Standard deviation in parentheses.

*9 farmers bought two varieties in both categories and are therefore included in both categories for the analysis of socio-demographics.

1 USD = 99.9 KES at the start of the data collection, 18 March 2019.

The socio-demographic profile of farmers preferring varieties of the three company categories is reported in Tables 5 and 6. Gender or age did not have an effect on farmer preferences. In Embu, farmers with larger maize fields preferred the varieties from IC. These were also the farmers who bought a larger seed quantity and sold a larger part of their harvest. In Kakamega, the farmers who purchased varieties from PDC were the ones that kept most of their harvest for their own consumption. Lastly, the key product attribute was explored for the different varieties. Overall, yield remains the most mentioned key attribute across the different categories. In Kakamega, early maturity stood out for PDC and IC to a lesser extent. In Embu, there was no difference in key attributes between IC and KSC.

## 5. Discussion and conclusion

Extensive investments have been made, and will likely continue to be made, in developing a strong and economically viable domestic maize-seed sector in maize-dependent countries in sub-Saharan Africa and beyond. With this study, the goal was to take stock of the presence of these PDC in Kenya and obtain more insights into their positioning versus the present IC and KSC. Investments in domestic seed production had a strong and positive effect on the availability of seed varieties coming from PDC, responsible for one third of the available products on the market. The variety release age was at par with the IC; both still had some older varieties in the market but were making investments to commercialize new maize varieties, unlike KSC, of which the most recent commercial variety was released in 2009. Under the assumption that genetic gains through breeding are higher for recently released varieties (Atlin, Cairns, and Das 2017), PDC and IC provided greater value to farmers. On the other hand, the low investments of those two company categories in late-maturing varieties, highly preferred in the main maize-producing zones in Kenya, resulted in passing over a significant customer base. Their quasi-monopoly on late-maturing varieties implied minimal competitive pressure on KSC to invest in developing and marketing new varieties; the parastatal can easily hold its dominant position with its current market offer.

KSC has one additional advantage: reliable and widespread farmer access to its products. While its strong position and dominance with older varieties have often been questioned (Smale and Olwande 2014; Das et al. 2019; Swanckaert 2012), the focus of discussion has mainly been on the product itself and not about access to those products. Our findings show that the parastatals’ strong position seems to be supported by product availability. They were present in all agro-dealers, with reliable access to its products, according to agro-dealers in our sample and lower prices than the competition while maintaining margins for retailers. While access being the strength of KSC, it seemed to be the domestic companies’ weak point. Not only were they not present in a large part of the agro-dealers, but reliability of supply also was not as good as that of KSC or the international competition.

Another key point of discussion in the competitor analysis was the affordability of the product. In that sense, Kenya represented a special situation with the presence of KSC and their fixed recommended price of 1.80 $/kg. ICs in Kenya have ignored this benchmark and have priced their seed products significantly higher at 2.50 $/kg, with relatively little variation on that price point. For domestic companies, data showed a greater variation in pricing strategy. Almost half of the seed was sold at the same benchmark price as the international companies, while the other half was sold below that price but almost none of them lowered to the KSC benchmark price. Price setting can be determined with respect to production cost and margin. If the production cost of PDC was lower compared with the IC (because of lower transportation costs), then PDC could make the same profit on a kg of seed while selling at a lower price. However, there was no evidence that this was the case, as ICs were likely to enjoy considerable scale in their breeding, production and marketing operations. More likely, seed pricing by PDC responded to their marketing strategy – a calculated effort to undercut the price of the international companies. However, farmers who explicitly wanted a cheap variety could have always gone for the well-known varieties offered by the parastatal that had stood the test of time. To the extent that price is an indicator of quality (Wolinsky 1983), setting seed prices lower than the competition in imperfect markets may likely send negative signals to farmers regarding seed quality. For a product, such as seed, where evaluation is mainly based on experience attributes (i.e., attributes that can only be evaluated after product use), price and brand are some of the few attributes to evaluate a product at the point of purchase. Another possible strategy for PDC could be to set sales price at the same level of the prices from ICs, but increase margins of retailers (resulting in higher retailer surplus) and use their vested interest and position to “push” their products to the farmer (Ailawadi et al. 2009; Rutsaert and Donovan 2020).

Looking at the customers, there were some differences among the buyers of KSC, PDC, and IC. The most consistent element was that farmers who purchased from IC sold a larger part of their seeds and in Embu, these were also the farmers with larger fields and purchase volumes. Another key element was the preference of farmers who purchased seed in rural and urban locations. Varieties of IC were selling much better in urban locations. In relation to that, farmers preferring those varieties were also willing to pay a higher travel cost. For IC, it did not seem to be necessary to extend their networks to rural zones. However, if the focus of PDC is on smallholders with reduced budgets (including travel budget), they should consider what the most cost-effective ways are to extend their networks to remote areas.

While the latest reports still confirmed KSC dominance in the Kenyan seed market (The African Seed Access Index 2016), keeping its position will become an increasingly difficult task if their material is judged to be inferior against the new products being released by PDC and IC. However, having the product alone will not automatically result in increased market share. PDC will need to invest in reliable access to seed, i.e., having a steady supply in their target areas, with backing from retail networks. Being in competition with IC, with larger budgets, on one hand, and a parastatal with undercutting prices, on the other hand, forces them to optimally invest their resources in high-value products supported by smart and targeted marketing investments (Heiman, Ferguson, and Zilberman 2020). Only the companies able to do this will be able to compete in the long run.

Access to improved seed for farmers will remain a key pillar of development programs and there is still a great need for investments in this field to increase the adoption of improved varieties in sub-Saharan Africa. However, when programs are focusing on replacement of current varieties that are not suitable to deal with climate change with a new generation of stress-tolerant varieties (Atlin, Cairns, and Das 2017), an assessment of the commercial potential of these new products should be an indispensable part of product development. Without a clear understanding of the farmer needs, an assessment of the competitor products or a good understanding of the competitive advantage of the new variety, most new market introductions are likely to fail. In 2017, CGIAR launched the Excellence in Breeding platform to unify breeding ideas, technology, resources, demand, and capacity across species and systems (Hickey et al. 2017). One of the key pillars of this platform is to mainstream and optimize product design and management by developing product profiles that can increase the success of product-replacement strategies. Actions like this can help to increase return on investments of modern breeding programs. However, it should not stop at improving the breeding programs. Research partners like National Agricultural Research Systems (NARS), CGIAR, and the donor community need to make sure that they not only supply those companies with top-quality material but also provide necessary support with respect to market intelligence, farmer and consumer preferences and best practices in product promotion and sales.

## Notes

1. Varietal release date is not the same as the date of commercialization. After release, the variety still needs to go through National Performance Testing (NPT) and bulking of parent seeds so it still can take 4 to 6 years before the variety is released.

2. Later correspondence with one of the key domestic seed companies in the dry zone informed that the company was struggling with seed production and therefore unable to produce sufficient quantities for the 2019 March–June season.

3. Exchange rate on 18 March 2019 (start of the field work) 1$ = 99.9 KES.
